# When Can Clades Be Potentially Resolved with Morphology?

**DOI:** 10.1371/journal.pone.0062312

**Published:** 2013-04-25

**Authors:** David W. Bapst

**Affiliations:** Geophysical Sciences, University of Chicago, Chicago, Illinois, United States of America; Monash University, Australia

## Abstract

Morphology-based phylogenetic analyses are the only option for reconstructing relationships among extinct lineages, but often find support for conflicting hypotheses of relationships. The resulting lack of phylogenetic resolution is generally explained in terms of data quality and methodological issues, such as character selection. A previous suggestion is that sampling ancestral morphotaxa or sampling multiple taxa descended from a long-lived, unchanging lineage can also yield clades which have no opportunity to share synapomorphies. This lack of character information leads to a lack of ‘intrinsic’ resolution, an issue that cannot be solved with additional morphological data. It is unclear how often we should expect clades to be intrinsically resolvable in realistic circumstances, as intrinsic resolution must increase as taxonomic sampling decreases. Using branching simulations, I quantify intrinsic resolution across several models of morphological differentiation and taxonomic sampling. Intrinsically unresolvable clades are found to be relatively frequent in simulations of both extinct and living taxa under realistic sampling scenarios, implying that intrinsic resolution is an issue for morphology-based analyses of phylogeny. Simulations which vary the rates of sampling and differentiation were tested for their agreement to observed distributions of durations from well-sampled fossil records and also having high intrinsic resolution. This combination only occurs in those datasets when differentiation and sampling rates are both unrealistically high relative to branching and extinction rates. Thus, the poor phylogenetic resolution occasionally observed in morphological phylogenetics may result from a lack of intrinsic resolvability within groups.

## Introduction

Assembling the tree of life is a premier task of modern biology, and morphology-based phylogenetic analyses are the main avenue for reconstructing relationships among extinct lineages. However, morphological phylogenetic analyses sometimes produce ambiguous results. For example, parsimony analyses of morphological data often find multiple conflicting topologies with the same parsimony score. Consensus summaries of these parsimony analyses collapse these conflicts into unresolved polytomies. Although molecular analyses also can find conflicting relationships, there remains a concern that morphological phylogenetics may be particularly inadequate for fully understanding relationships among taxa [1]. Poorly resolved relationships in morphological analyses are often blamed on data quality issues, such as non-independence among characters due to correlated evolution [2], the difficulty of defining and selecting reliable homologs [3] or the incompleteness of data matrices [4]. Regardless of the explanation given, the implication is that more morphological characters or increased taxonomic sampling will help resolve these relationships. Conflicting relationships might represent groups that cannot be further resolved into a dichotomous hierarchy, such as ‘hard’ polytomies formed by simultaneous speciation events [5], but real non-dichotomous relationships are assumed to be rare and thus polytomies are generally considered failures to resolve.

Unresolved polytomies can also be produced when morphological data are incapable of providing further resolution, such that no amount of character information provides true synapomorphies to unite the descendants of a branching event, despite the group being a real clade (i.e. sharing a common ancestral population to the exclusion of other taxa). This phenomenon has been noted previously in the context that sampling ancestral lineages or multiple descendants of a single ancestor generates apparent polytomies in cladistic analyses [6], [7], [8], [9], [10], [11]. The relationships and sampling of morphologically defined taxa (‘morphotaxa’) can produce a lack of opportunity for character change, and thus a lack of synapomorphies for resolving lineages into dichotomous groups. A lack of synapomorphies produces phylogenetic uncertainty regardless of whether analyzed phylogeny is reconstructed under a parsimony, likelihood or Bayesian framework. As such groups lack the intrinsic potential to be resolved via actual synapomorphies, irrespective of the actual morphological data collected, I refer to this property as whether a particular dichotomous clade is ‘intrinsically’ resolvable. The intrinsic resolution of an entire dataset is the proportion of potentially resolvable clades, assuming that identifiable synapomorphies occur where any opportunity exists.

The primary intent of this study is to test whether intrinsically unresolvable clades are expected with realistic sampling. Although the potential for clades to lack intrinsic resolvability has been long recognized when morphotaxa are completely sampled through time [9], [11], intrinsic resolution is rarely suggested as an explanation for poorly resolved phylogenies, perhaps because it is unclear how frequently such phenomena should be expected in actual datasets. Incomplete taxon sampling should reduce the probability of sampling ancestors or multiple descendants of the same ancestor, and thus increase intrinsic resolution. However, it is unclear how poorly sampled the fossil record must be to avoid groups lacking intrinsic resolution, or whether sampling only extant lineages at some time-slice could generate datasets with high intrinsic resolvability.

I use stochastic branching simulations of the fossil record to measure intrinsic resolution of datasets under various scenarios of morphological differentiation and sampling, including when only extant taxa are sampled. Rather than directly simulate morphological characters, I only model differentiation events and measure resolvability as the proportion of true clades of sampled morphotaxa that share at least one differentiation event to the exclusion of other sampled morphotaxa. This methodology isolates losses of resolvability due only to the intrinsic lack of opportunities for accruing potential synapomorphies at differentiation events. The degree of intrinsic resolution measured in these simulations is the maximum possible degree of resolution. Real character data and tree reconstruction can only further decrease the number of true resolvable clades by introducing factors such as homoplasy and character state reversals. Adding these components to these simulations would obscure the intent to quantify intrinsic resolution, which is independent of actual character data.

Simulations can shed light on what circumstances of sampling and differentiation are most likely to produce clades lacking intrinsic resolvability. Simulation-based estimates of intrinsic resolution can also serve as a basis for defining tree priors for paleontological phylogenetic analyses [11]. However, magnitude is not the only interesting quality of intrinsic resolution. We often conceptualize ancestor-descendant relationships as attributes of lower-level taxa, such as species. As intrinsic resolution is a result of the pattern of ancestor-descendant relationships among morphotaxa, it might be assumed that unresolvable clades are more likely to occur among individual low-level taxa instead of major clades. If this were true, a lack of intrinsic resolution should create polytomies which are particularly shallow on the tree or contain only a small number of child branches. In addition to quantifying magnitude of intrinsic resolution, simulations can also be used to test if intrinsically unresolvable clusters show any bias towards the size or depth of clades.

### Intrinsic Resolution and Categorizing Patterns of Morphological Differentiation

The concept of intrinsic resolution assumes that the same discrete morphological characters used to define, distinguish and reliably recognize taxon units across space and time are the same set of characters available for phylogenetic analyses. Under such a framework, a change in one or more characters causes that lineage to be immediately recognized as a new morphotaxon. Such morphological transitions must be infrequent in groups which have long species-level durations [12], [13], [14], [15] and these shifts appear to be geologically instantaneous, although some exceptions occur in highly sampled records [16]. It is this apparent finite potential for character change events, i.e. differentiation events, that leads to the ‘intrinsic’ lack of an opportunity for true synapomorphies to accrue, regardless of the number of morphological characters sampled.

**Figure 1:**
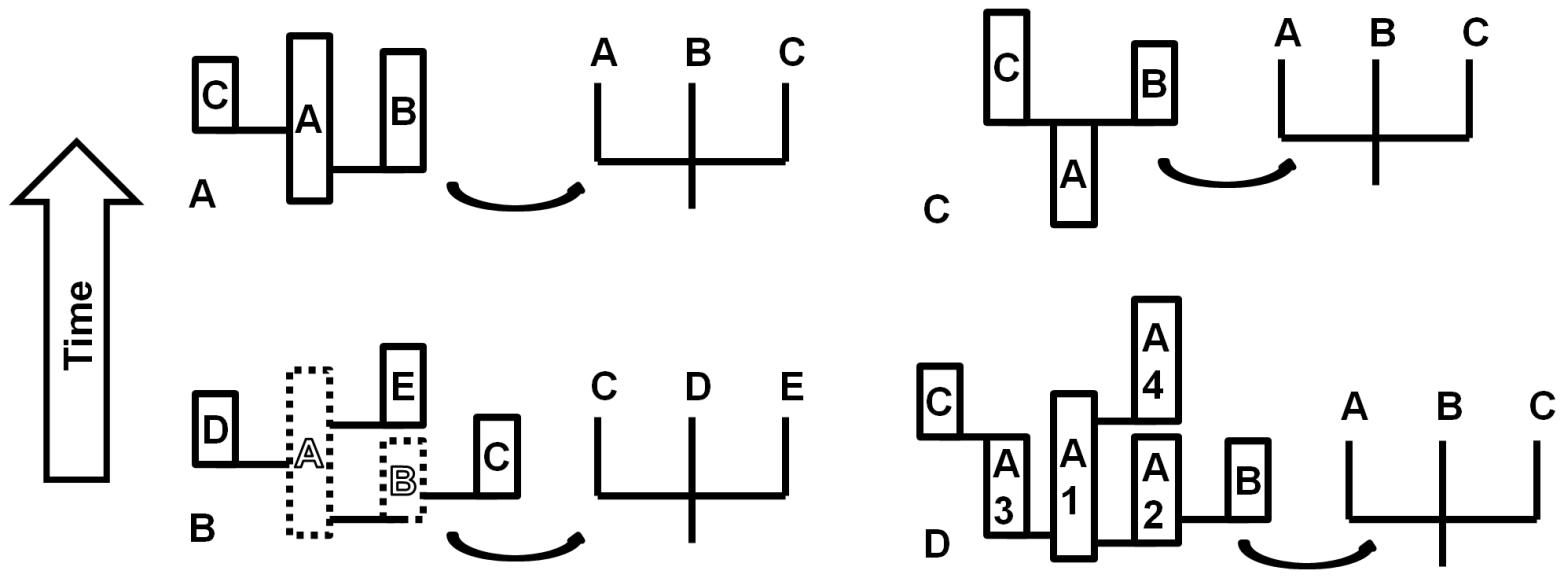
The sampling and differentiation of lineages impacts the potential to resolve clades. These diagrams depict how patterns of morphological differentiation and sampling of ancestors impact the distribution of potential synapomorphies and thus the intrinsic capability to resolve clades with morphological data. In (a), as there is no morphological change over the duration of the ancestral taxon A, there can be no true synapomorphies which would unite any pair of the sampled taxa (ancestor A or its descendants B and C) to the exclusion of a third. In (b), the taxa with a dashed outline (taxa A and B) have not been sampled and are not included in the supposed cladistic analysis. However, even though A and B are ancestral, the sampled taxa are all descended from the same persistent ancestor and thus no synapomorphy exists to produce an additional nested clade. This causes the node to be intrinsically unresolvable. In (c), under a bifurcating cladogenesis pattern, polytomies are only produced when the ancestor A is sampled, as each morphotaxon can only have two descendants. The fourth example (d) is an example with cryptic cladogenesis and anagenesis, where the formation of several sampled descendants from a cryptic ancestral complex of multiple undifferentiated lineages produces an unresolvable set of relationships. The taxa in (d) labeled A1, A2, A3 and A4 are undifferentiated cryptic lineages, which would be identified as a single morphotaxon ‘A’ for the purposes of taxonomic assessment and phylogenetic analyses.

The magnitude of intrinsic resolvability depends on the patterns of both morphotaxon sampling and differentiation events across lineages [9]. For any set of three or more morphotaxa, relationships among them will not be intrinsically resolvable if either of two conditions is satisfied. First, if that set includes the common ancestral morphotaxon of the other taxa (Figures 1a and 1c) or, second, if all taxa in the set are descended from a single persistent morphotaxon (Figure 1b). Ancestry may be direct or indirect (*sensu* Foote [17]) to satisfy these conditions; i.e. sampled descendants do not need to be the immediate daughter taxon of the ancestral morphotaxon. Thus, while sampling ancestral taxa and their descendants always results in intrinsically unresolvable polytomies, taxa can be intrinsically unresolvable even without sampling any ancestors (Figure 1b). As with hard polytomies, intrinsically unresolvable clades can be reframed conceptually as instances in which the assumption that the number of clades (or branches) present for some number of taxon units is less than that expected for an entirely binary tree [11], [18].

**Figure 2:**
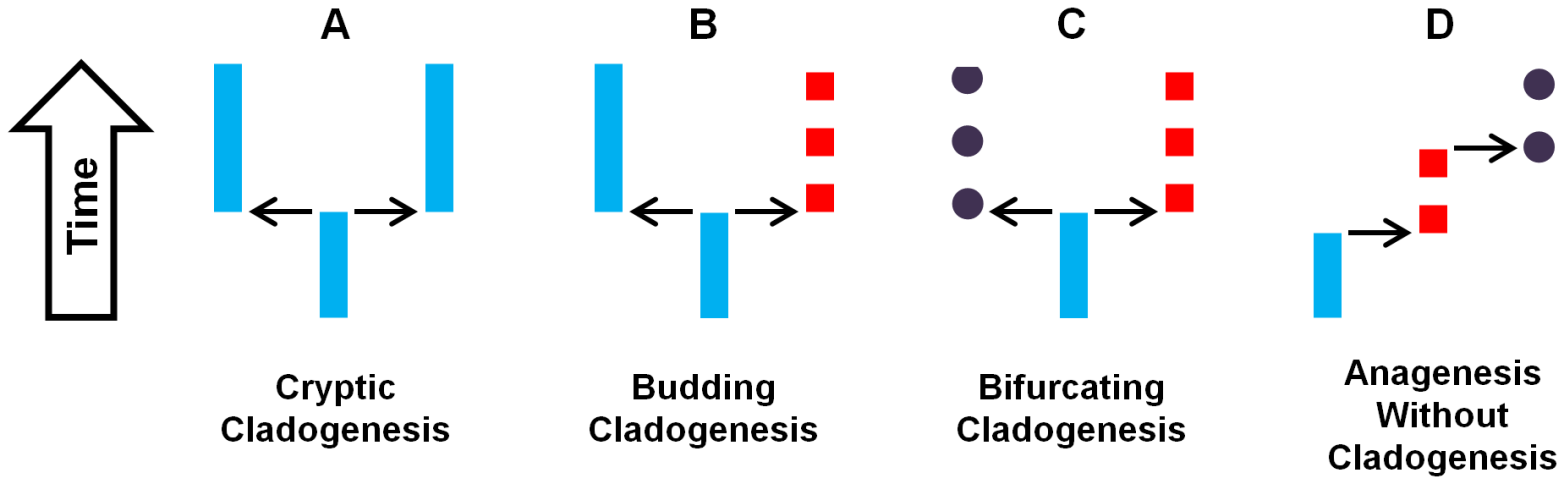
Four models of morphological differentiation. If differentiation occurs instantaneously, there are three possible models of how differentiation may evolve at branching events (cladogenesis). From left to right: (a) under cryptic speciation the two daughter lineages are morphologically identical to their ancestor; (b) with budding cladogenesis, a single daughter lineage is undifferentiated from the ancestor while the other daughter is distinguishable as a separate morphotaxon based on systematic characters; (c) bifurcating cladogenesis produces two daughter lineages which are both recognizably different relative to their ancestor. The fourth pattern of differentiation shown here is repeated events of anagenesis, which is differentiation unassociated with branching. Branching is depicted identically across these figures to contrast the morphological patterns alone and does not reflect discontinuity of populations.

Models of morphological change have been previously categorized under different ‘speciation’ types [9], [17], [19]. In order to conceptually distinguish the production of new lineages (i.e. branching events or cladogenesis) from the production of new morphologically distinguishable taxon units, more commonly referred to as speciation, instead changes in morphology will be referred to as ‘morphological differentiation’ events. (Differentiation remains equivalent to speciation if we define speciation as the origin of new distinguishable morphotaxa, but this interpretation may not agree with some commonly used species concepts [20].) A number of classifications have been applied to differentiation patterns; a system similar to Foote’s [17] is used here (Figure 2). Morphological differentiation events within a lineage that are unassociated with branching events are referred to in this paper as anagenesis (Figure 2d), sometimes also called pseudo-speciation. Cladogenesis events, when lineages branch and increase in number, have been traditionally further classified in paleontological literature based on their relationship with differentiation events. Considering only branching events that produce two daughter lineages, there are three possible patterns of differentiation at cladogenesis. These are ‘cryptic’ cladogenesis, ‘budding’ cladogenesis and ‘bifurcating’ cladogenesis, depending on whether none, one or both resulting lineages become morphologically distinguishable from the ancestral morphology (Figure 2a–c). Under budding and cryptic cladogenesis, not all resulting lineages change morphologically at speciation events, such that ancestral morphotaxa persist through the branching event without any apparent change. The three cladogenetic patterns and anagenesis can be combined to describe a wide variety of morphological differentiation models.

The phenomenon of intrinsic unresolvability of clades has not been assessed in simulations of cryptic cladogenesis, where no morphological differentiation occurs at branching, forming two daughter lineages indistinguishable based on morphology. This is an important model to consider given evidence of cryptic species being common in some groups [21], [22] and some sister lineages persisting with no differentiation over tens of millions of years [23]. Theoretically, we would expect frequent cryptic branching to generate complexes of numerous indistinguishable cryptic lineages, that would appear to be some smaller number of long-lived morphotaxa from a taxonomist’s perspective. Thus, cryptic cladogenesis would be expected to behave similarly to budding cladogenesis in rendering clades unresolvable (Fig. 1d). To realistically mimic the treatment of unknown cryptic lineages in morphology-based analyses, cryptic lineages in this study are always collapsed into morphologically distinguishable taxonomic units on modeled cladograms.

Using simulations of branching patterns and differentiation events, I perform two analyses. In the first, I compare how the resolvable proportion of clades varies across a broad array of differentiation and sampling patterns, including incomplete sampling in the fossil record and sampling of only extant morphotaxa. However, it is important to consider simulations in which both anagenetic and sampling rates are varied, as high rates of anagenesis relative to cladogenesis can produce high intrinsic resolution independently of sampling. This is particularly relevant for estimating the expected intrinsic resolution of real data, as we often do not have estimates of the anagenetic rate of differentiation [but see 9]. In the second analysis, simulations are conducted at different rates of anagenesis and sampling to test which scenarios produce datasets that can both (a) match empirical observations from the fossil record and (b) have a high intrinsic resolvability.

## Results

### Size-constant versus Clade-constant Simulations of the Fossil Record

Using branching simulations of morphotaxa under different patterns of differentiation and sampling, we can quantify and compare the expected magnitude of intrinsic resolvability under each scenario. One hundred simulated clades were generated and sampled for each combination of thirteen differentiation models and four sampling models. Intrinsic resolution of simulated datasets was quantified as the intrinsically resolvable proportion of clades, the proportion of sampled clades potentially resolvable with shared synapomorphies (see Methods).

**Figure 3:**
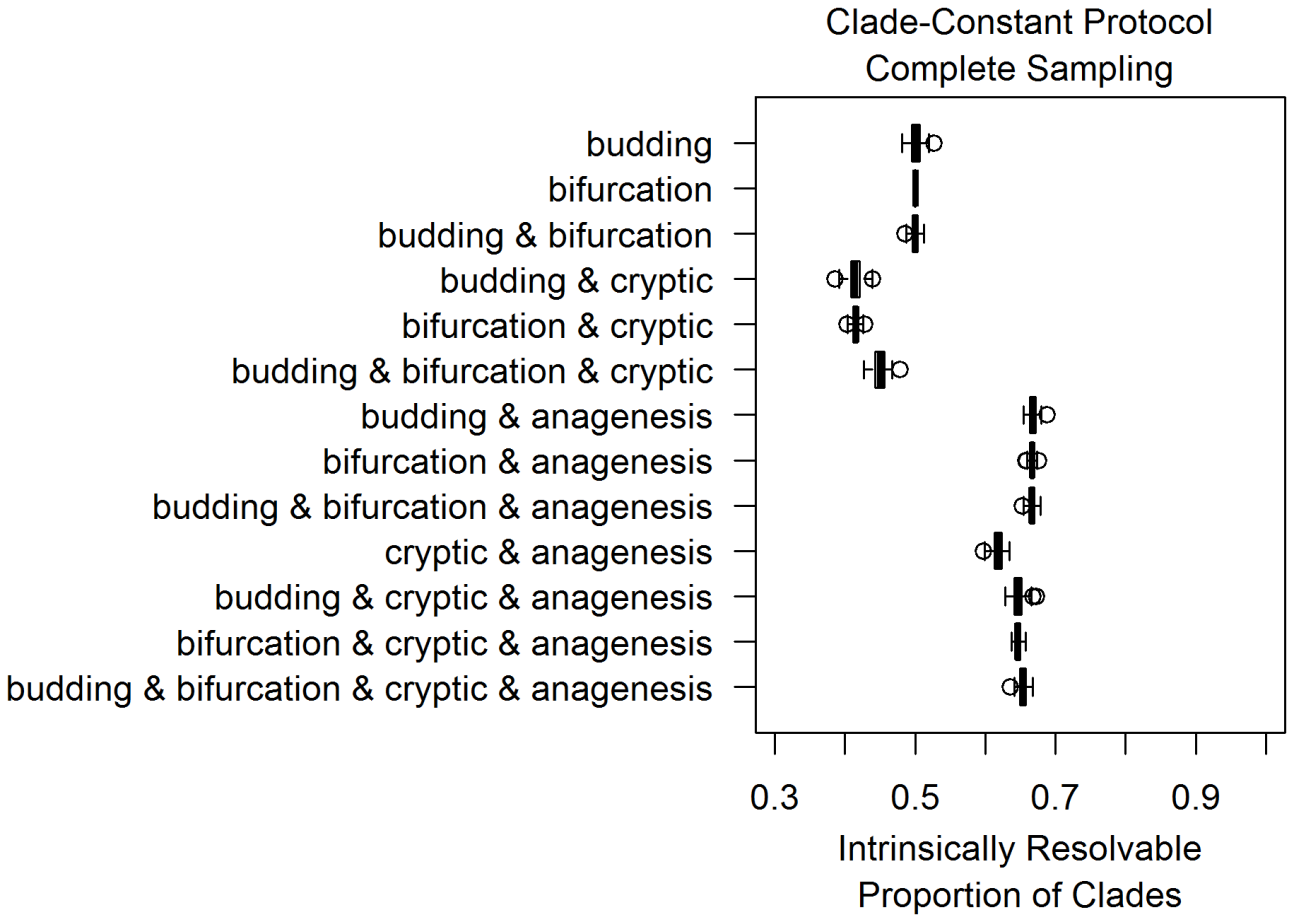
Under complete sampling, the resolvable proportion of clades is low under all models of differentiation. The thirteen boxplots in this figure are based on thirteen models of morphological differentiation, listed on the left. Each boxplot represents measurements of the resolvable proportion of clades for 100 simulations performed for each differentiation pattern under complete sampling. Simulations were conditioned to have one hundred sampled taxa on average at a sampling rate of 0.01 per Ltu, under the clade-constant protocol discussed in the methods.

**Figure 4:**
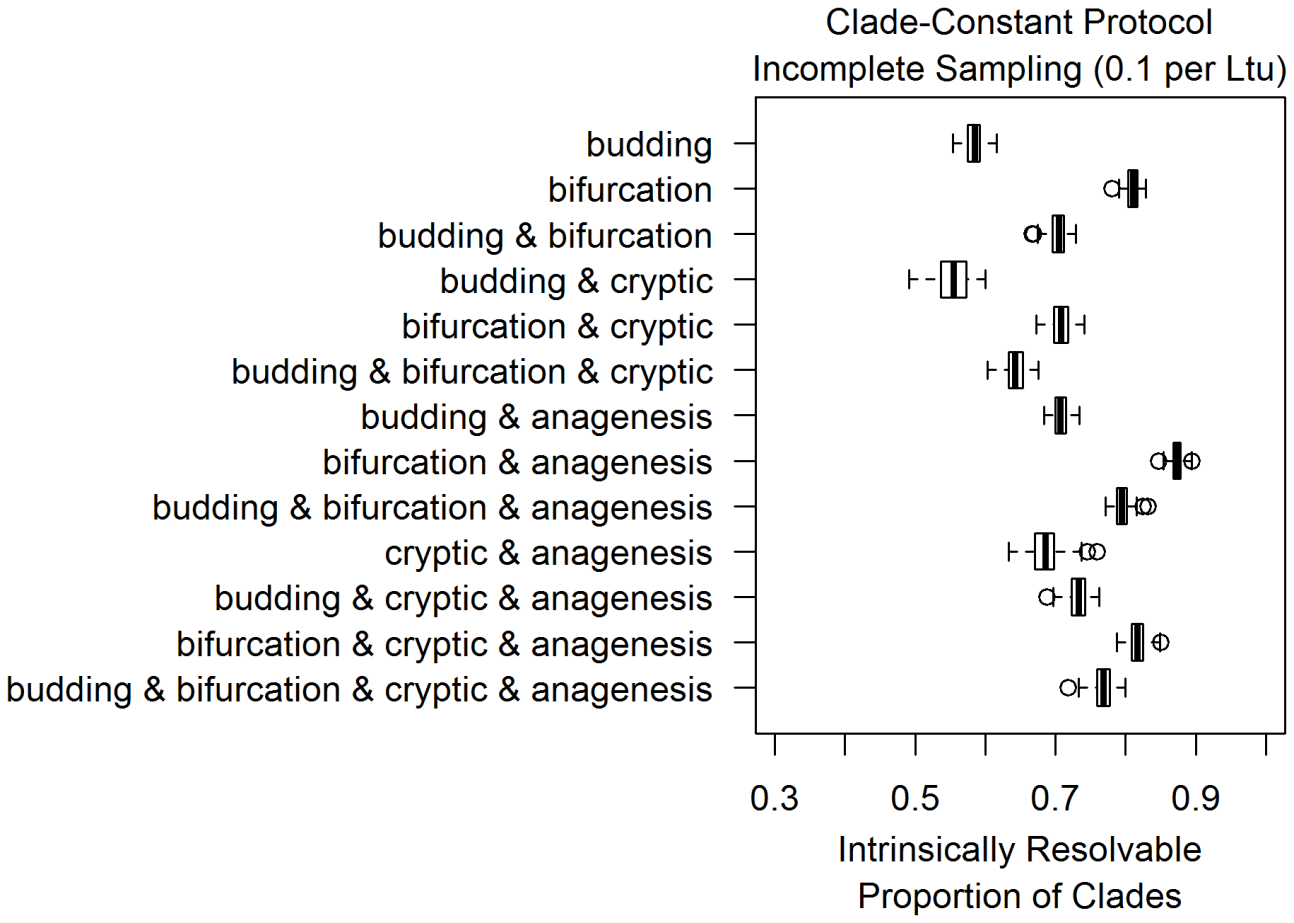
The resolvable proportion under each differentiation model increases as sampling rate decreases to 0.1 per Ltu, but not evenly across all differentiation models. As in figure 3, the thirteen boxplots in this figure are based on thirteen models of morphological differentiation, listed on the left. Each boxplot represents measurements of the resolvable proportion of clades for 100 simulations performed for each differentiation pattern under incomplete sampling, with a sampling rate of 0.1 per Ltu. Simulations were conditioned to have one hundred sampled taxa on average at a sampling rate of 0.01 per Ltu, under the clade-constant protocol discussed in the methods.

**Figure 5:**
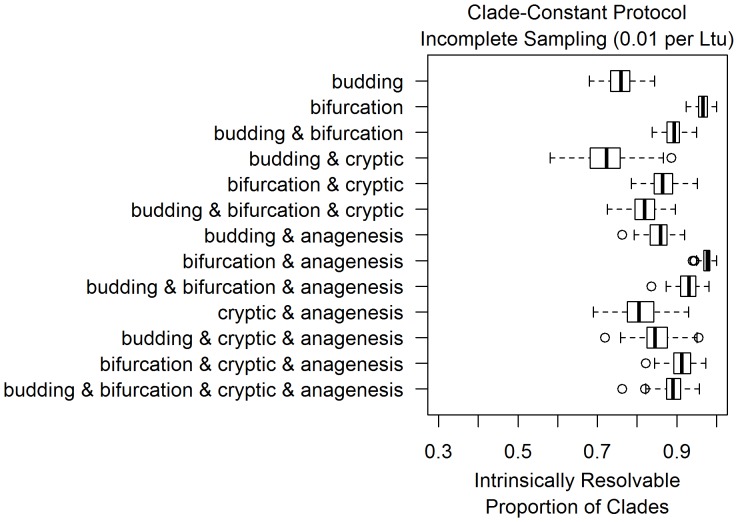
At a very low sampling rate (0.01 per Ltu), some but not all models of differentiation predict that almost all clades will be resolvable. As in figure 3, the thirteen boxplots in this figure are based on thirteen models of morphological differentiation, listed on the left. Each boxplot represents measurements of the resolvable proportion of clades for 100 simulations performed for each differentiation pattern under incomplete sampling, with a sampling rate of 0.01 per Ltu. Simulations were conditioned to have one hundred sampled taxa on average at that sampling rate, under the clade-constant protocol discussed in the methods.

To test whether simulation conditioning affected results of this analysis, analyses emulating the fossil record were performed across different models of sampling under either (a) a size-constant protocol, where the number of sampled taxa was kept nearly equal, or (b) a clade-constant protocol, where the total number of original morphotaxa was kept constant. For each combination of differentiation pattern and sampling scenario, these two protocols produced very similar mean estimates of the resolvable proportion of clades (Figures 3, 4, 5 and S1, S2, S3). This similarity suggests that simulation estimates of intrinsic resolvability are robust to variation in the number of original or sampled morphotaxa, despite the large difference in the number of sampled morphotaxa. For example, simulated cladograms with complete sampling had one hundred morphotaxa under the size-constant protocol and several thousand morphotaxa under the clade-constant protocol. Thus, the intrinsically resolvable proportion of clades is controlled by differentiation pattern and sampling frequency, and appears to be independent of both the number of morphotaxa considered and the total amount of evolutionary history present in a dataset.

The major difference in intrinsic resolution between these two protocols is the dispersion of simulation values. Under the clade-constant protocol, which often generated datasets with high numbers of sampled morphotaxa when sampling was relatively high, the observed resolvable proportion was much more tightly clustered around the mean value for that simulation set. The difference in the dispersion of the resolvable proportion values suggests that as the number of sampled morphotaxa increase (and thus the number of sampled clades), the resolvable proportion of clades converges on a single value dependent on simulation parameters. The consistent estimation in a stochastic simulation implies that a set probability of intrinsic resolvability may exist for a given model of differentiation and sampling. If so, trees of larger size have more precise values of resolvable proportion just as larger samples of binomially-distributed tests provide more precise estimates of the underlying success probability. A simple comparison finds that the observed variance in resolvable proportion within simulation sets is effectively predicted by the expected variance under a binomial distribution (R^2^ of 0.942 for simulation sets from both size-constant and clade-constant protocols). This relationship supports the hypothesis that there is a fixed probability of intrinsic resolution for each set of simulations. It should be feasible in future work to deterministically model the probability of intrinsic resolvability given model parameters for sampling and differentiation, thus providing informative tree priors for phylogenetic analyses of fossil morphotaxa [11].

### Contrasting Differentiation Patterns across Sampling Scenarios

Figures 3, 4 and 5 display simulation results across combinations of differentiation and sampling models under a clade-constant protocol, as those estimates of intrinsic resolvability are much less dispersed due to their larger sample size. Corresponding plots from size-constant protocol simulations are included in the supplementary (Figures S1, S2, S3). When extinct clades are fully sampled in simulations (Figure 3), there is a relatively low degree of intrinsic resolvability of clades with about half of all clades being intrinsically unresolvable. As would be expected on theoretical grounds, the inclusion of cryptic cladogenesis lowers intrinsic resolvability while the presence of anagenesis increases the resolvable proportion of clades. Among simulations with anagenesis as a component, sixty to seventy percent of clades are intrinsically resolvable.

Incompletely sampled fossil records have higher intrinsic resolution than the completely sampled scenarios, with resolvability increasing as sampling rates decreased from 0.1 per lineage *time-units (henceforth, per Ltu) to 0.01 per Ltu (Figures 4 and 5). This is the expected consequence of decreased taxonomic sampling, leading to differentiation events being more frequently present on the phylogenetic branches separating sampled morphotaxa. This increased frequency of differentiation events between taxa directly leads to an increased probability that any given clade will be intrinsically resolvable with morphology. The increase in the resolvable proportion varies considerably across differentiation patterns as sampling became more incomplete. For example, although the addition of anagenesis events produces a similar resolvability increase in multiple patterns under complete sampling (Figure 3), those same differentiation models appear to have distinct probabilities of intrinsic resolvability under incomplete sampling (Figures 4 and 5). This effect is attributable to the details of how clades become intrinsically unresolvable under each pattern. As bifurcating cladogenesis only produces intrinsically unresolvable clades if ancestral morphotaxa are sampled, simulations containing bifurcation increase more quickly in resolvability as sampling becomes more incomplete.

Incomplete sampling models at 0.1 per Ltu are comparable to sampling probabilities estimated for groups of shelly marine invertebrates (Table 1; [24]). Simulations at this degree of sampling predict a range of one half to one tenth of all potential clades in each dataset being intrinsically unresolvable. Even the model with the highest proportion of resolvable clades, bifurcation with anagenesis, does not produce any simulation datasets that are completely intrinsically resolvable with morphology. Models such as budding cladogenesis and cryptic cladogenesis with anagenesis are on the low end of this spectrum. Under an order of magnitude lower sampling rate (0.01 per Ltu), these same differentiation patterns predict that twenty to thirty percent of clades are intrinsically unresolvable while most patterns predict higher levels of resolution (Figure 5).

**Table 1:** Sampling regimes used in simulations of the fossil record, listed by sampling rate, with equivalent sampling probabilities and completeness.

Instantaneous Rate of Sampling(per Ltu)	Probability of Sampling a Taxon At Least Once Per Time Unit	Expected Taxonomic Completeness	Expected Waiting Time Between Sampling Events (for a Single Lineage)
Infinite (complete sampling)	1	100 %	0 time-units
0.1	0.095	50 %	10 time-units
0.01	0.0099	9 %	100 time-units

Equivalent values for other common sampling parameters are calculated with respect to other simulation parameters. The sampling probabilities and completeness ignore effects resulting from cryptic cladogenesis. Sampling probabilities also assume that taxa fully extend through single intervals of a single time interval.

**Figure 6:**
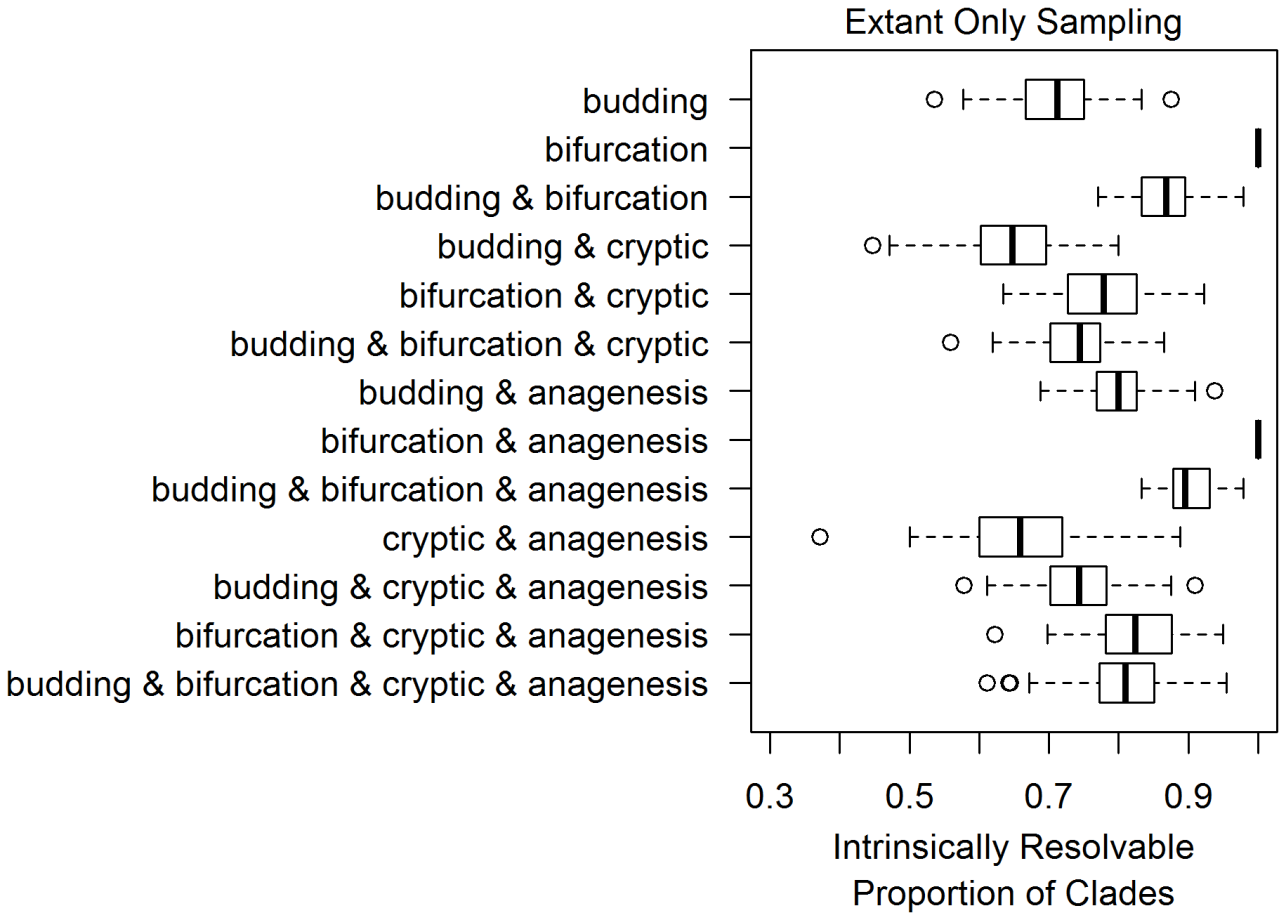
Datasets composed only of living morphotaxa are expected to have fewer resolvable clades than datasets from poorly preserved fossil records, under most differentiation models. As in figure 3, the thirteen boxplots in this figure are based on thirteen models of morphological differentiation, listed on the left. Each boxplot represents measurements of the resolvable proportion of clades for 100 simulations performed for each differentiation pattern, with between 50 and 300 co-extant morphotaxa sampled.

Relative to the previous simulations, datasets with 50 to 300 living taxa had larger ranges for resolvable proportion under every differentiation pattern (Figure 6). This increase in variance may result from the relatively fewer morphotaxa sampled in each simulation, leading the observed resolvable proportion of each simulation to be more variable. Under most differentiation models, the obtained values of resolvable proportions are lower than those obtained in the poorly sampled fossil record simulations (with a sampling rate of 0.01 per Ltu). Models where all cladogenesis is bifurcating are an exception to this, being fully resolvable. This is because ancestral taxa are necessary for clades to become intrinsically unresolvable under bifurcation, but ancestral taxa by definition cannot persist to the present in a bifurcation-only model. Thus, issues of intrinsic resolution are likely relevant to morphological phylogenetics of only living taxa, unless morphological differentiation is predominantly bifurcating.

The observed maximum sizes of intrinsic polytomies differ considerably across conditioning protocols in these simulations. The largest intrinsic polytomies had many more daughter lineages in clade-constant simulations relative to their equivalent size-constant simulations, reflecting the much larger number of sampled morphotaxa. Within the size-constant simulations of fossil record-like datasets, the largest polytomies contained about five daughter lineages on average, for all sampling models and across all thirteen differentiation patterns. However, as these values are per-simulation maxima, their distributions do not behave like normal distributions and show a significant right skew, with a long tail toward very large numbers. A few outlier simulations had as many as twenty or thirty daughter lineages in their largest polytomies. The extant-only simulation also had maximum polytomy size distributed close to five, except for differentiation patterns with pure bifurcating cladogenesis as these had no polytomies (Figure 6). However, as polytomy size must be considered relative to the number of sampled morphotaxa and given most extant-only datasets had slightly more than fifty sampled morphotaxa, this implies that the largest intrinsically unresolvable polytomies in extant-only datasets might be as much as double in size relative to paleontological datasets.

There was little evidence that intrinsically unresolvable clades are shallower or deeper situated in cladograms than intrinsically resolvable clades. Chi-squared tests of the association between tree depth and the frequency of polytomies for each simulation produce p-values distributed relatively homogenously between zero and one, as would be expected for test comparisons on randomly distributed data. Some combinations of differentiation patterns and sampling scenarios could not be evaluated, as they consistently produced cladograms where every node was an intrinsic polytomy (bifurcation under complete sampling) or where no nodes were polytomies (bifurcation with only extant morphotaxa sampled). Of particular significance is the simulations of extant-only cladograms, as all sampled taxa are separated from the root by equal amounts of time at their time of observation. In this set of simulations, only ∼3% of simulations had statistical p-values less than 0.05 and none were statistically significant when these values were Bonferroni corrected for multiple comparisons. Considered along with the analyses of maximum polytomy size, I conclude that intrinsically unresolvable relationships can occur at any hierarchical location in a cladogram and could produce intrinsic polytomies with a relatively large number of daughter lineages, in both datasets of fossil and living morphotaxa.

### The Interplay between Differentiation Rate and Sampling Rate

**Figure 7:**
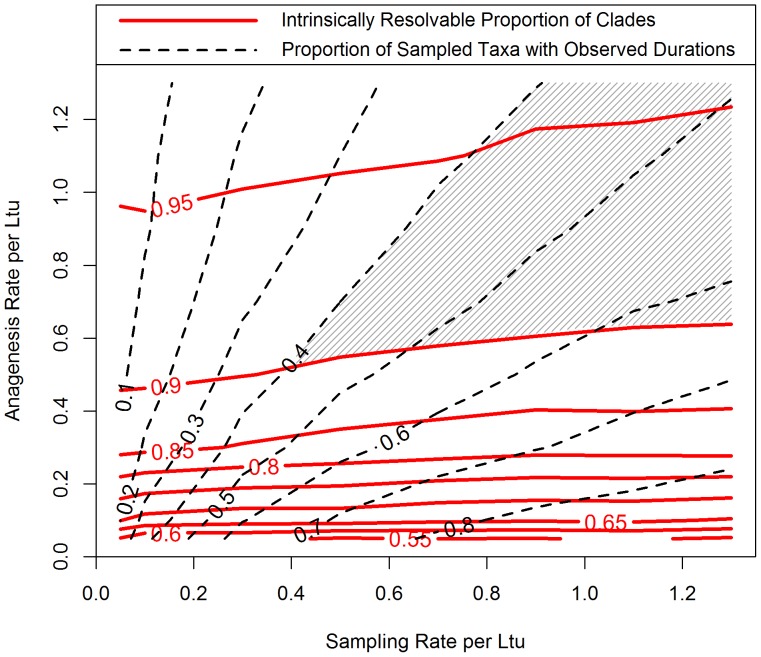
Clades which that match the fossil record in taxon durations require both high anagenesis and sampling rates to have high intrinsic resolution. Simulated extinct clades were generated under cryptic cladogenesis with anagenesis, with both varying sampling and anagenesis rates. For each combination of rates, 100 clades were generated, conditioned to have 100 sampled morphotaxa on average. The mean resolvable proportion of clades and the mean proportion of taxa with observed durations at each combination were used to generate two overlaid topographic contours. The shaded region represents the combinations of anagenesis and sampling rates which produce both highly resolvable clades (at least 0.9 intrinsically resolvable) and clades with realistic proportions of taxa with observed durations (at least 0.4 of sampled taxa). Branching and extinction rates were held constant at 0.1 per Ltu.

If anagenesis rates are high relative to branching rates, sampled sets of taxa may be highly resolved but not match empirical observations of the fossil record, such as the persistence of morphotaxa through several geologic intervals. By comparing the resolvable proportion of clades and the proportion of taxa with an observed duration across different combinations of sampling and anagenesis rates, we can test what parameter combinations generate datasets which match our observations of well-sampled fossil records and have high degrees of intrinsic resolvability. Under a single differentiation pattern, cryptic cladogenesis with anagenesis, differentiation only occurs at anagenesis events and thus the anagenesis rate is exactly equivalent to the rate of differentiation. By taking the mean proportions measured for sets of simulations at each combination, two topographical landscapes can be mapped onto a bivariate space of sampling rate versus anagenesis rate (Figure 7). A comparison of these two surfaces reveals that intrinsic resolvability is predominantly controlled by the rate of differentiation events, with resolvability increasing with the differentiation rate. As sampling rate increases, there is only a slight upward shift in the contours, reflecting a slow decrease in the resolvable proportion. This weak relationship implies that datasets can be highly resolved at high sampling rates, as long as rates of differentiation are equally high. This finding differs from the above (i.e. Figures 3, 4, 5), which found that sampling could have a large effect. However, this is probably a false contrast as those previous simulations did not contrast the effects of sampling and differentiation (the frequency of differentiation was held constant). The proportion of morphotaxa sampled more than once depends on both rates, with more taxa having durations in geologic time when sampling rate is high and the anagenesis rate is low.

A further step for assessing these proportions is to set credibility cutoffs and consider those combinations of rates which produce simulated datasets similar to our observations of the fossil record and have a high resolvable proportion of clades. Foote and Raup [25] present range data for morphological species from several groups, including trilobites, bivalves and mammals. These range data are given in discrete temporal intervals, ranging from 60 feet of stratigraphic section to geological intervals of five million years. Among these datasets, approximately forty to seventy percent of species appear in more than one interval. A limited set of rate combinations produce datasets expected to have more than forty percent of taxa with observed durations and where nine out of ten clades are intrinsically resolvable (Figure 7, shaded region). The 0.9 cutoff for resolvable proportion of clades is arbitrary, but topographic contours reveal that the expected resolvable proportion drops quickly as the rate of anagenetic differentiation decreases. Under a model of cryptic cladogenesis and anagenesis, it is necessary that both differentiation and sampling rates must be high relative to diversification rates to satisfy our prior criteria.

**Figure 8:**
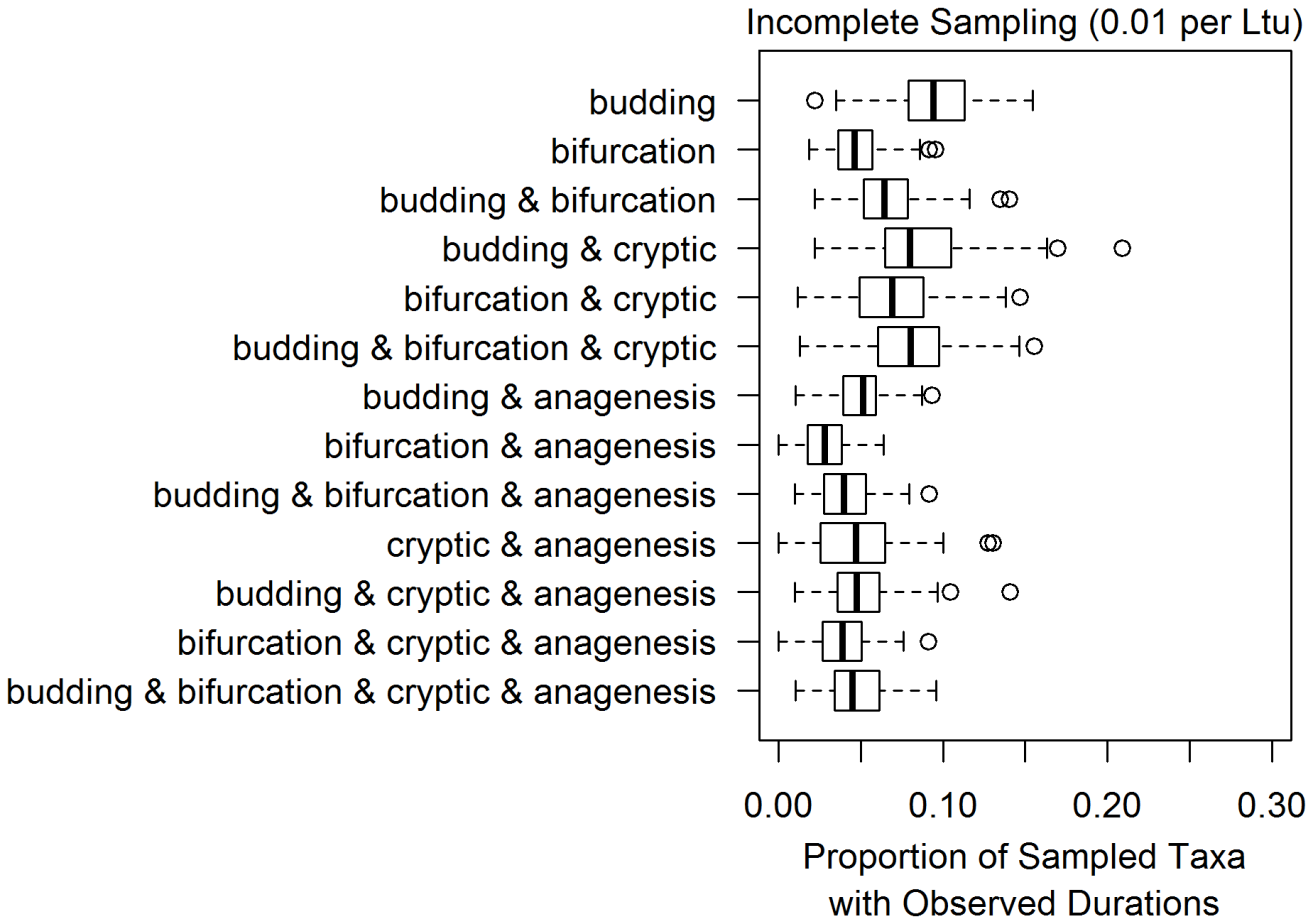
At very low sampling rates, none of the differentiation patterns generate realistic frequencies of taxa with observed durations. The thirteen boxplots in this figure are based on thirteen models of morphological differentiation, listed on the left. Each boxplot represents measurements of the resolvable proportion of clades for 100 simulations performed for each differentiation pattern under incomplete sampling, with a sampling rate of 0.01 per Ltu. Simulations were conditioned to have one hundred sampled taxa on average at that sampling rate.

It is conceivable that other differentiation patterns could provide different answers. Above, simulations conducted at low sampling rates (0.01 per Ltu) found that some patterns had relatively high intrinsic resolvability (Figure 5). To test whether these differentiation patterns at this low sampling rate also produced realistic proportions of taxa with observed durations, I conducted a further set of simulations. For each of the thirteen possible differentiation patterns, one hundred extinct clades were generated and their durations sampled at a rate of 0.01 per Ltu. These simulations were conditioned to have a hundred sampled morphotaxa on average. At this low sampling rate and across the thirteen models of differentiation considered, less than thirty percent of sampled taxa were sampled more than once on average (Figure 8). Thus, the simulations of differentiation patterns at low sampling rates described above cannot be considered realistic scenarios for paleontological data.

## Discussion

### Constraints on High Rates of Differentiation and Sampling

This study finds that high rates of differentiation and sampling are necessary to produce simulated datasets which match *both* the empirical observation of persistent taxa in some fossil records *and* have a high degree of intrinsic resolution (Figure 7). Such high rates relative to diversification rates are probably unrealistic. The minimum required anagenesis rate is five times higher than the rates of branching or extinction. In such a scenario, only one out of six apparent morphotaxon origination or termination events in the fossil record would actually represent cladogenesis or extinction. Instead, the majority of observed events in the fossil record would be pseudo-speciation and pseudo-extinction events, disconnected from usual diversification processes. This is contrary to a common assumption in the paleobiological literature that taxonomic appearance rates reflect diversification rates [26] or that the duration of taxa reflect their respective extinction susceptibility [27], [28]. Although there is little empirical work quantifying the frequency of anagenetic differentiation to actual branching events in the fossil record, it has been long recognized that anagenesis must be less frequent than cladogenesis for morphotaxa to coexist with their apparent descendant taxa [12].

Stronger evidence against these high-rate scenarios comes from evaluating the necessary high sampling rates, which are much higher than many empirical measures of sampling frequency [24], [25], [29]. In addition, it is probable that the shaded region of Figure 7 is also unlike the fossil record in another property: the length of the observed durations. These simulations were conducted in continuous time and, consequently, many of the taxa with observed durations may have short observed durations, due to the high anagenesis rates. If these simulations had been conducted on a realistic time-scale composed of discrete intervals each with considerable length, only a small proportion of sampled morphotaxa would likely be observed within multiple intervals.

Given the lower likelihood of high rates of differentiation and sampling, it is difficult to explain the existence of well-sampled fossil records, where morphotaxa extend across multiple intervals, without also needing to account for clades that potentially lack intrinsic resolution. While it was previously recognized that unresolved clades in morphological analyses could be due to sampled or persistent ancestors, these simulation analyses suggest that a lack of intrinsic resolvability should be fairly common in real datasets. If these simulations are accurate reflections of reality, workers should not generally expect all potential clades in a morphological dataset to be intrinsically resolvable.

### Fundamental Assumptions of the Simulation Framework

All simulation analyses must be judged on the basis of their primary assumptions. The basic concept behind the differentiation models used here is that morphological character change occurs only at rare differentiation events, which seems reasonable given the existence of persistent morphotaxa in the fossil record. Character data is assumed to be a closed system utilized for both defining morphotaxa and constructing phylogenies. If a clade is intrinsically unresolvable, it will be such regardless of the effort made to collect character data within such a closed system. In other words, collecting more character data from the same information used to define morphotaxa will not change intrinsic patterns of resolution, not if morphotaxa can persist for any measurable geologic duration. This is a fundamental and inescapable limitation of morphology just like the recent finding of common non-independent character change [2]. Instead, increasing the number of morphological characters in a dataset is more likely to lead a worker to incorrectly resolve intrinsically unresolvable polytomies. As the number of characters increases, lineages in an unresolvable cluster are more likely to independently acquire convergent character states which will appear to be homologous, similar to the phenomenon of long branch attraction [30]. Molecular data are exempt from the assumptions of intrinsic resolution as those characters aren’t used to define morphotaxa. Most morphological datasets consist of many fewer characters than molecular datasets, reflecting the relatively small amount of information available for defining morphotaxa.

While considerable effort is made in this study to consider a variety of morphological differentiation patterns, only a single model of sampling in the fossil record is considered. Under this model, sampling events are equally likely along any lineage, at any point in time. Real sampling scenarios are probably more complex, and some models have been formulated which account for those complexities [31], [32]. In order to produce datasets that both are highly resolvable with morphological characters and can satisfy the observation of long-lived undifferentiated morphotaxa, a sampling model would need to fulfill several criteria. Such a model would require a sampling bias against ancestral taxa, such that sampled lineages are predominantly descendents from ancestral lineages in regions with particularly poor preservation rates. To contain both long-lived morphotaxa and to have high intrinsic resolvability, sampled descendant lineages would also need to be unlikely to produce their own sampled descendants, either by anagenesis or cladogenesis. These terminal descendent taxa would also need to be resistant to extinction, so that undifferentiated lineages are sampled in multiple geologic intervals. Although a diverse range of sampling models is discussed in the paleobiological literature, none seem likely to match these caveats. One model satisfying these conditions is a scenario where diversification and morphological change predominantly occurs in tectonically inactive settings where weathering and reworking of sediment can dominate over burial. This disagrees with work that found correlations between diversification and tectonic active regions [33] and coral reef occupancy [34], both being environments that are well represented in the fossil record. Thus, the assumptions about sampling made for these simulations appear to be reasonable with respect to the addressed question.

The simulations in this paper treat morphotaxa as units that are not further dissected on the basis of morphology, even when composed of multiple cryptic lineages. These taxon units do not need to be the minimally definable morphospecies used in the fossil record, which often cannot be further divided based on discrete traits. My choice of the word ‘taxa’ throughout this paper is deliberate: if a phylogenetic analysis considered family-level taxa and did not further dissect those taxon units, that scenario would equally match the assumptions of this paper and I expect that the results of this study can scale to higher taxonomic levels as used in many studies. The model of cryptic cladogenesis used in this study is inspired by Patzkowsky’s [35] model of ‘paraclade’ diversification. The differentiation pattern with budding and cryptic cladogenesis and no anagenesis is functionally identical to Patzkowsky’s description, where branching may produce new within-taxon lineages or produce entirely new supraspecific taxa [35]. In those cases where supraspecific taxa are represented as single taxonomic units in cladistic analyses and their monophyly has not been previously verified, the structure of relationships among such higher-level taxa should be very similar to differentiation patterns with cryptic cladogenesis. Furthermore, the supposition that issues of intrinsic resolution might be avoided by using supraspecific taxa can be rejected given the finding in this study that intrinsic resolution does not vary systematically over a cladogram. If clades lacking intrinsic resolvability are present, such unresolvable relationships are just as likely to be found among three recently diverged morphospecies as among three great clades diverged by hundreds of millions of years.

### Testing for a Lack of Intrinsic Resolution in Real Datasets

Testing between hypothesized explanations for an observed amount of phylogenetic uncertainty in empirical datasets (i.e. polytomies on a consensus tree) is a considerable challenge. Unfortunately, there is no simple test to identify whether actual observed polytomies result from intrinsic unresolvability. An ideal approach would be to compare the degree of cladistic resolution in a real dataset with estimates of intrinsic resolvability, via simulations parameterized with estimates of sampling intensity and differentiation patterns. Unfortunately, very few empirical measurements of differentiation patterns exist. Wagner and Erwin [9] found support for budding cladogenesis over other differentiation patterns, but these findings were partly based on the distribution of polytomies within their analyzed phylogenies. Modeling benchmark values of intrinsic resolvability for real datasets requires somehow obtaining independent estimates of differentiation pattern.

Additionally, the number of polytomies observed in actual cladistic datasets cannot be easily related to the specific phenomenon of intrinsic resolvability. Intrinsically unresolvable clades are generated by a specific combination of taxon sampling and morphological differentiation processes, while conflicting hypotheses of relationships observed in real datasets can also be produced by other phenomena, such as the character reversal, homoplasy and poor character definitions. In addition, the choice of consensus method affects the number and type of polytomies that appear, e.g. the difference between majority rule consensus trees and strict consensus trees. Comparing empirical datasets directly to simulated values of intrinsic resolution could only be used to determine if an observed amount of phylogenetic uncertainty is consistent with a lack of intrinsic resolvability. Furthermore, simulation-based estimates would have wide confidence intervals when applied to datasets with a small numbers of taxa.

A number of issues must be resolved to attempt any comparison of observed and expected degrees of phylogenetic uncertainty. A clear metric of phylogenetic uncertainty in real datasets is needed, as different consensus methods give different numbers of polytomies. Differentiation patterns in empirical data must be distinguished, requiring the diagnosis of ancestor-descendant relationships [9]. One common test is to treat taxa as ancestral if such taxa emerge as plesiomorphic in a prior cladistic analysis [8], with no derived autapomorphies. However, this test may be overly zealous in rejecting ancestral relationships, as character reversal can give ancestral taxa apparent autapomorphies [18], [36]. Similarly, a wide range of equally-valid alternative ancestor-descendant relationships may exist for a given set of stratigraphic occurrences of morphotaxa, depending on the degree of sampling. Most phylogenetic inference methods that simultaneously consider character and stratigraphic occurrence data explicitly allow for reduced numbers of clades in the resulting tree; i.e. allowing for intrinsically unresolvable clades [18], [37], [38]. The best option for distinguishing between differentiation patterns would be Bayesian methods to fit models of character change and sampling simultaneously to available data, allowing for the estimation of ancestor-descendant relationships [11]. Although the simulations in this study are a step toward producing the tree priors needed for such an approach [11], such analyses are not yet fully developed at present, and thus it is unclear at present how to test between alternative hypotheses for phylogenetic uncertainty.

### Implications of the Expected Magnitude of Intrinsic Resolvability

This study reveals that there is a considerable extent within realistic sampling scenarios under which large numbers of intrinsically unresolvable clades can be obtained. The few quantitative analyses of morphological differentiation patterns have mainly found evidence for budding cladogenesis, such as Wagner and Erwin’s analyses using datasets of planktonic foraminifera and Paleozoic gastropods [9]. Given that simulations with budding cladogenesis have a low resolvable proportion of clades relative to other examined differentiation patterns, Wagner and Erwin’s results [9] imply a high potential for intrinsically unresolvable clades in morphological phylogenetics. In simulations of relatively well-sampled clades of fossil taxa with budding cladogenesis, only sixty percent of clades were intrinsically resolvable on average (Figure 4).

The simulations conducted here suggest that the highest intrinsic resolution should occur in those clades with the poorest sampling. While this may seem counterintuitive, it stems from intrinsic resolution being a property of whether differentiation events occur along the branches separating clades of sampled taxa. More incompletely sampled datasets have more evolutionary history separating taxa and thus a higher frequency of differentiation events along branches. However, this does not mean that cladograms of poorly sampled groups should be expected to be well-resolved, as intrinsic resolution is only one component of the resolution obtained in actual phylogenetic inference. Wagner [39] conducted realistic maximum parsimony analyses of simulated fossil clades with fully simulated character data and found that inferred relationships further decreased in accuracy as sampling of the simulated fossil records decreased, due to difficulties distinguishing homology from homoplasy with a limited number of character states. In addition, strict parsimony analyses by Wagner preferred incorrectly resolving polytomies due to homoplastic character convergence, even though polytomies were allowed in output trees [39]. This aspect of parsimony caused tree reconstruction in simulations under budding cladogenesis to be more inaccurate than with corresponding simulations of bifurcating cladogenesis. This agrees with previous work [19] which found that extant-only datasets had low phylogenetic accuracy when generated under a model of ‘punctuational’ character evolution (i.e. budding cladogenesis). Thus, at very low sampling, a higher proportion of clades are intrinsically resolvable but the clades inferred may be inaccurate. Higher sampling intensity provides the best accuracy for reconstructing intrinsically resolvable clades, at the cost of more clades lacking intrinsic resolvability.

The resolvable proportion of clades did not differ greatly between simulations of a well-sampled fossil record and simulations of extant-only morphotaxa. Unless bifurcating cladogenesis has been the predominant mode of differentiation (contrary to [9]), a lack of intrinsic resolution should also be present in datasets composed only of modern taxa. Increased sampling of either fossil or extant taxa should decrease the expected intrinsic resolvability of a dataset. Interestingly, this hypothesized relationship may have already been tested. Cobbett et al. [40] randomly removed single taxa from forty-five real morphological datasets and compared the change in the number of most parsimonious trees found, as a metric for the degree of uncertainty in the phylogenetic topology. Despite fossil taxa having more incomplete morphological data, the removal of extinct or extant taxa produced similar decreases in the number of most parsimonious trees [40]. This observation that decreased sampling of either extinct or extant taxa produce similar increases in phylogenetic certainty is predicted if uncertainty is predominantly a result of sampling intrinsically unresolvable clades.

Given the results of this study, intrinsic unresolvability should be considered a valid explanation for poorly resolved relationships in morphology-based phylogenetics, although one of many alternatives. The frequency of clades lacking intrinsic resolvability under realistic sampling scenarios (Figures 4 and 6) suggests that such issues could be a leading factor of phylogenetic uncertainty in groups with well-sampled fossil records.

Literature on consensus methods often stresses that consensus diagrams should not be used in analyses of evolutionary pattern, as the resulting branching diagram is an incomplete summary of a sample of trees [41]. Some workers using maximum parsimony datasets have advocated using the sample of most parsimonious trees as the basis for phylogenetic comparative analyses, instead of a single consensus topology with polytomies [42]. However, if we expect that conflicting relationships are occasionally generated by a lack of intrinsic resolvability, then the placement of polytomies on a consensus diagram can actually convey valuable information about evolutionary pattern. For example, without polytomies, it might be difficult to reconstruct that several sampled morphotaxa share a single persistent ancestor. This information is not available if only a sample of most parsimonious trees is evaluated, unless the analysis somehow also considers node support across the entire input sample of trees. While no ideal consensus method exists for identifying phylogenetic uncertainty resulting from intrinsic resolution, we should consider the possibility in phylogenetic analyses of macroevolution, particularly in groups with well-sampled fossil records.

If poorly resolved morphological cladograms do reflect the actual obtainable set of relationships, the poor phylogenetic resolution of some morphological datasets may simply reflect a historical pattern of differentiation that obscures the ability to reconstruct groupings using shared derived characters. Researchers should treat polytomies as potentially natural and expected elements in a morphology-based analysis, even if it is difficult to distinguish between a lack of intrinsic resolvability and other factors. If maximum parsimony is biased against identifying conflicting support for relationships [39], it may be warranted to take a conservative approach and collapse unsupported branches in cladistic analyses [39], [43]. Poorly resolved phylogenies may reflect only a lack of morphological information necessary to resolve that clade, a deficit of evolutionary pattern that cannot be corrected by adding more morphological characters. In those circumstances where a lack of intrinsic resolution is expected a priori, such as groups with well-sampled fossil records, poorly resolved phylogenies probably represent more realistic estimates of relationships than completely resolved phylogenies, which may inadvertently result from convergence. Although phylogenetic uncertainty is not more likely to represent intrinsically unresolvable clades than any other factor, intrinsic resolvability should not only be considered an explanation of last resort. If even as many as ten percent of clades in an analysis lack the intrinsic capability to be resolved with morphology, unresolved polytomies cannot be considered only blemishes on a morphological phylogeny. Instead, polytomies should be treated as potential indicators of the complex interaction between taxon-sampling and the historical process of character changes across lineages.

## Methods

### Basic Structure of Simulations: Diversification and Differentiation

For all simulations in this study, lineages are stochastically simulated using a conditioned simulator that generates sequences of events in continuous time. Cladogenesis, extinction and anagenesis events are treated as ‘competing’ Poisson processes occurring independently along lineages, with each process having its own instantaneous rate parameter. This matches the standard model for birth-death process, which treats branching and extinction as competing Poisson processes [44], [45]. Under competing Poisson processes, waiting times between events are exponentially distributed, with a mean waiting time equal to the reciprocal of the sum of the rates for all competing Poisson processes. The probability that the next event is a particular event type is the rate of that process divided by the sum of all rates. By randomly drawing waiting times from such a distribution and sampling event types with their probabilities as weights, the diversification of lineages can be quickly and effectively simulated in continuous time. In the particular output produced by the simulator used here, a matrix of temporal first and last occurrences and morphological identities is given for each lineage, rather than each morphotaxon. This allows for the possibility of lineages which are morphologically indistinguishable from their ancestor or other sister lineages (i.e. cryptic complexes of lineages).

Although these simulations are performed on an arbitrary continuous timescale, ranges of parameter values are chosen from empirical studies so simulator time-units are comparable to millions of years, the time-unit frequently used in paleobiology. For all simulations in this paper, rates of diversification, differentiation and sampling were held constant within simulation runs. The instantaneous rates of cladogenesis and extinction are set equal at 0.1 per Ltu. Estimates of speciation and extinction rates have a wide range, but the values used here match measurements for modern marine invertebrate groups [14], [21], [46]. By holding rates of branching and extinction equal, there is a sufficient probability of generating large clades prior to complete extinction, allowing us to simulate datasets similar to those observed for extinct groups in the fossil record. This assumption is also realistic, as speciation and extinction rates are nearly equal on long timescales in the fossil record [14], [46].

In addition to the simulation of cladogenesis and extinction, the birth-death simulator is integrated with models of morphological differentiation, such that any of the four basic differentiation patterns or any mixture of those patterns can be simulated simultaneously with the diversification processes. Anagenesis is treated as a competing Poisson process with its own instantaneous rate parameter, just like cladogenesis and extinction. Each time anagenesis occurs, the lineage is considered a new morphologically distinct taxon unit from its ancestor (which becomes pseudo-extinct). At branching events, daughter lineages may undergo differentiation under the selected type of cladogenetic differentiation pattern. Both daughter lineages differentiate if bifurcating cladogenesis occurs (thus causing the ancestor to become pseudo-extinct), only one of the two daughter lineages differentiate under budding cladogenesis, and neither daughter lineage differentiates under cryptic cladogenesis.

Simulations of diversification and differentiation are performed with the function *simFossilTaxa* from the software library *paleotree* [47]. Each simulation run begins with a single lineage, followed by the stochastic selection of an event type and a waiting time to that event using the competing Poisson model. If an event is cladogenesis, the type of cladogenesis is selected based on the input probabilities of cladogenesis being budding, bifurcating or cryptic. If the first lineage is not yet extinct, another waiting time and event is drawn for the same lineage until either extinction occurs or the lineage survives beyond the maximum time constraint for the simulation (for this study, 10^∧^6 time-units was used for simulations of the fossil record). Waiting times and events are drawn for the second lineage to originate, then the third and so on. The simulation is evaluated between drawing individual waiting times to check if various pre-set stopping criteria are met, such as a particular number of co-extant lineages. Generated datasets that match stopping criteria are accepted or rejected based on an additional set of acceptance criteria dependent on the simulations, such as whether all lineages have gone extinct for simulations necessitating datasets of only fossil taxa (e.g. Figs 3, 4, 5). Accepted simulations are output as tables recording the birth, death and transformation of morphologically differentiated and undifferentiated lineages (and, thus, also contain information on the timing of all branching events).

### Simulating Incomplete Sampling and Conditioning on Numbers of Sampled Morphotaxa

The degree of sampling in the fossil record is one of the most important aspects of these simulations. Although sampling is sometimes quantified as the probability of sampling a lineage at least once per time interval or as the expected proportion of all taxa sampled [25], [29], these statistics are partly dependent on the rates of origination and extinction [26]. Sampling events are modeled as a time-constant Poisson process, matching previous work [29], [48] and matching the treatment of cladogenesis, extinction and anagenesis events. These sampling simulations are secondarily performed on simulation datasets output by the diversification and differentiation simulator, via the *paleotree* function *sampleRanges*. Provided with the per-morphotaxon times of origination and termination, sampling events are dropped into the true temporal ranges of the taxa by stochastically drawing serial waiting times between sampling events. These serial waiting times are drawn from an exponential distribution determined by the input sampling rate. Waiting times are accrued from the start of a taxon’s range (i.e. its origination) until the summed waiting times are greater than the taxon’s range (i.e. the last sampling event occurs after the taxon’s termination time). This approach relies on the useful memory-less attribute of the exponential distribution, such that the waiting times from a taxon’s origination to the first sampling event for that taxon should be identically distributed to the waiting times between sampling events. If the first waiting time drawn is greater than a taxon’s entire range, such that the first sampling event occurs after a morphotaxon terminates, then that taxon was simply never sampled. For sampled taxa, the first observed appearance date of each taxon is the first sampling event to occur after a taxon true time of origination and the last observed appearance date is the last sampling event to occur before a taxon’s termination time. If these are the same sampling event, than that taxon was only sampled once and has no observable duration.

Simulations with incomplete sampling of morphotaxa require special conditioning in order to produce final datasets containing some desired average number of sampled taxa. These simulations must be conditioned on the total number of ‘original’ morphotaxa present prior to simulating sampling. When diversification, anagenesis and sampling are treated as time-constant Poisson processes, the relationship between the total number of original morphotaxa and sampled morphotaxa is described by the equation *N = D/(r/(r+k))*, where *N* is the average number of original morphotaxa necessary, *D* is the desired average number of sampled morphotaxa, *r* is the instantaneous rate of sampling per Ltu and *k* is the instantaneous rate of morphotaxon termination per Ltu. The denominator is simply the probability of sampling any single morphotaxon at least once [25]. The morphotaxon termination rate is the frequency per Ltu of morphotaxa either going extinct or undergoing morphological differentiation, so as to be unrecognizable (and thus pseudo-extinct). Across the range of differentiation models considered in this paper, the instantaneous rate of a morphotaxon terminating is equal to the sum of the rates of extinction, anagenesis and the product of the branching rate with the probability of cladogenesis being bifurcation. Although the equation above predicts the necessary average number of original morphotaxa, the distribution of sampled morphotaxa for a clade with some number of original morphotaxa is not symmetrically distributed around the mean. To obtain a desired number of sampled morphotaxa on average, simulated datasets are only accepted with a minimum of *N* original morphotaxa and a maximum of *2N*. In practice, this routine produces simulated datasets with total numbers of sampled morphotaxa distributed around the desired average. When simulations include cryptic lineages, single morphotaxa may be composed of multiple lineages and the termination rates calculated using the above equation can only be a rough approximation. However, despite violating model assumptions, the described conditioning routine functions adequately in the simulations with cryptic lineages to produce final datasets with a given number of sampled morphotaxa.

### Measuring Intrinsic Resolution for Simulations

Simulation results can be translated into a nested hierarchy by treating the origin of each new differentiable morphotaxon (i.e. each differentiation event) as the origin of a new intrinsically-resolvable clade. The transformation function *taxa2cladogram* from *paleotree* produces an ‘idealized’ cladogram, a rooted topology with no branch lengths. Branching nodes on these cladograms represent groups of lineages with the potential to share morphological synapomorphies (i.e. an intrinsically resolvable clade). Under the models of instantaneous differentiation considered here, morphological shifts among lineages are the only occasions at which new synapomorphies can arise and, thus, are the only information required to calculate which clades are intrinsically resolvable. Multiple undifferentiated lineages belonging to the same cryptic complex are collapsed to a single morphotaxon before acquiring the idealized cladogram. This collapsing of multiple cryptic lineages is meant to emulate the treatment of cryptic complexes as single morphotaxa as expected in any non-molecular study.

The resolvable proportion of clades is measured from the ideal cladogram as Colless’s consensus fork index [49]. This is the ratio of the number of observed clades (i.e. the number of branching nodes on the ideal cladogram) minus one (the root), divided by the total number of clades when all branching is binary, calculated as the number of sampled differentiated morphotaxa minus two. This metric is unaffected by the nesting structure among nodes. Colless originally proposed this metric for comparing the relative resolution of consensus trees, but the cladograms examined here are ideal representations of the relationships among lineages and not consensus diagrams. Thus, in this work, this metric is referred to as the intrinsically resolvable proportion of clades.

### Analyses under Various Differentiation Patterns and Sampling Rates

To compare the impact of differentiation patterns on intrinsic resolution of clades, simulated datasets are generated under thirteen combinations of the four basic differentiation patterns (listed in Figures 3, 4, 5, 6). The differentiation pattern with pure cryptic cladogenesis and no anagenesis is not considered, as only a single morphotaxon can ever exist in such a model. In patterns that include anagenesis, the rate of anagenesis is set equal to the 0.1 per Ltu value used for the diversification rates. When combinations of cladogenesis event types are simulated, their probabilities are set equal to each other (e.g. under a model with budding, bifurcating and cryptic cladogenesis, the probabilities of each type were one third). These parameter values are chosen solely to provide insight into the relative behavior of these combinations and may not be realistic parameter values. The equal proportions of cladogenesis types are only chosen to illustrate how such a mixture behaves in these simulations. Unfortunately, there are few empirical estimates related to differentiation patterns, such as the instantaneous rate of anagenetic differentiation. For each combination of one of the four sampling models and thirteen differentiation models, one hundred clades of a given number of taxa were simulated and transformed into idealized cladograms, from which statistics such as the resolvable proportion of clades were calculated.

The first three simulation sets are designed to mimic data from the fossil record and conditioned to accept only clades that went fully extinct due to stochastic processes. For their respective models of sampling, the first analyzes ideal cladograms with complete taxon sampling, the second is an incomplete sampling scenario with an instantaneous sampling rate similar to empirical estimates for shelly marine invertebrates [26], [31], and the third uses a sampling rate an order of magnitude lower than the second. The sampling rates used here can be transformed to more familiar sampling variables [25], such as the probability of sampling once per interval and the expected proportion of taxa sampled at least once, i.e. taxonomic completeness (Table 1). The estimates of sampling probability reported in Table 1 are rough transformations that assume that taxa fully extend through time intervals [26]. The minimum sampling rate used here is 0.01 per Ltu, although it is theoretically plausible that sampling rates may be much lower relative to diversification rates [17]. However, this would also require very large clades, potentially with millions of original morphotaxa represented by only a handful of sampled taxa. It is unlikely under a time-constant extinction rate for such enormous clades to go extinct stochastically within a computationally reasonable time-span; additional phenomena such as mass extinction events are necessary to drive such a group completely extinct. The 0.01 per Ltu minimum sampling rate used here is close to this simulation limit.

All simulations composed only of extinct taxa (i.e. the first three sampling models; Figures 3, 4, 5) are conducted under two different conditioning routines for the number of sampled morphotaxa. The first simulates all clades for each sampling model independently, conditioned to produce a hundred sampled morphotaxa on average in each simulation. In this way, the number of sampled morphotaxa, i.e. the cladogram size, is kept constant across simulations with different models of sampling and differentiation (thus ‘size-constant’). The second routine simulates a single large extinct clade with conditioning to produce a hundred sampled morphotaxa on average in simulations under the low sampling rate (the third sampling model). This very large clade is saved and re-used for the other two sampling models, producing idealized cladograms that encompass many more sampled morphotaxa. Under the complete sampling model, simulated cladograms contain thousands of morphotaxa. Thus, while the number of taxa are very different across the sampling simulations, the original clade datasets used are kept constant (hence, ‘clade-constant’). Simulating under both the size-constant and clade-constant routines allows us to consider possible artifacts arising from conditioning on either different numbers of original or sampled morphotaxa.

The fourth sampling scenario is intended to emulate a neontological study of morphotaxa. For each combination of differentiation patterns, one hundred clades were simulated until they reached a point in time where they had between fifty and three hundred living distinguishable morphotaxa, with simulations terminated immediately before the ‘next event’ (e.g. cladogenesis, anagenesis, etc) once sufficient living morphotaxa were obtained. This range was selected as it was difficult, under some differentiation models, to condition simulations to have a minimum of one hundred co-extant morphotaxa without also producing simulation runs that ‘exploded’ in diversity, resulting in computationally taxing large numbers of lineages. As with all simulations involving cryptic cladogenesis in this study, complexes of morphologically indistinguishable lineages are collapsed to a single lineage prior to producing the idealized cladogram. This mimics the information that would be available to a systematicist working on the phylogenetic relationships of several living morphotaxa using only morphological characters.

In addition to the resolvable proportion of clades, the depth and size of each intrinsically unresolvable polytomy is measured for each simulated dataset under a particular combination of sampling and differentiation models. The maximum number of branches in any polytomy on the idealized cladogram is recorded to quantify the upper size of intrinsically unresolvable clusters. The relationship between depth and the presence of polytomies in the idealized cladogram is also recorded, with branching node height measured as the number of previous nodes between a node and the root. To test for a relationship between node height and whether a given node was a polytomy, nodes on cladograms were divided into four height categories, with categories positioned to have similar numbers of nodes. The p-value of a chi-square test on the proportion of nodes in each category that are polytomies versus dichotomies is used to test for a potential relationship between height and the presence of polytomies.

### Comparing the Rate of Sampling against the Rate of Differentiation

The simulations above assess how combinations of sampling and differentiation patterns affect the intrinsic resolvability of clades, but do not address how intrinsic resolvability changes as sampling and differentiation rates are varied independently. Morphological change occurred simultaneously with cladogenesis in a majority of considered differentiation patterns, which confounds the impact of differentiation with diversification. The model where all cladogenesis events are cryptic and differentiation only occurs via anagenesis separates diversification from differentiation, allowing the rate of differentiation to vary while holding the rate of lineage diversification fixed. Using this differentiation model, a final set of simulations generates fully extinct clades under varying combinations of instantaneous sampling and anagenesis rates. Rates for both processes were varied between 0.05 and 1.3 per Ltu, with one hundred simulated datasets produced for each combination of sampling and anagenesis rates. Although 0.05 is higher than the minimum 0.01 per Ltu sampling rate used above, it was computationally difficult to produce simulation datasets across the entire range of anagenesis rates at a sampling rate of 0.01 per Ltu. The 0.05 per Ltu minimum used for this set of analyses is a compromise, while still being much lower than most estimates of sampling intensity for marine invertebrate records (Table 1, compare to [24]). Simulations were conditioned to produce one hundred sampled taxa on average, similar to the size-constant conditioning protocol described above. As with the simulations described earlier, the resolvable proportion of clades is calculated for each simulated dataset via the idealized cladogram.

In addition to examining how sampling and differentiation change the resolvable proportion of clades, the distribution of morphotaxon durations is also assessed for each simulation. At least in some fossil records [25], a relatively large proportion of morphotaxa are persistently recognized across geologic time-spans. Some combinations of sampling rate and anagenesis rate create scenarios where very few morphotaxa are sampled more than once, because differentiation events will be frequent and sampling events infrequent. To consider this aspect, the proportion of morphotaxa with an observed duration are measured and compared to empirical observations from the fossil record. For each set of one hundred simulations generated for each combination of sampling and anagenesis rates, I calculated the mean resolvable proportion of clades and the mean proportion of morphotaxa sampled more than once.

The functions employed here are written for the programming environment R [50] and included in the software library *paleotree* [47], dependent on the software library *ape* [51]. All of the above software is freely available from the Comprehensive R Archive Network. Simulated data values and specific scripts to reproduce analyses and figures described in this manuscript are submitted to Dryad and located at doi:10.5061/dryad.25131.

## Supporting Information

Figure S1 **Simulations of resolvability under complete sampling with size-constant conditioning protocol.** The thirteen boxplots in this figure are based on thirteen models of morphological differentiation, listed on the left. Each boxplot represents measurements of the resolvable proportion of clades for 100 simulations performed for each differentiation pattern under complete sampling. Simulations were conditioned to have one hundred taxa on average, under the size-constant protocol discussed in the methods. Compare to figure 3, which depicts the same analyses under the clade-constant protocol.
(TIF)

Figure S2 **Simulations of resolvability under incomplete sampling (rate of 0.1 per Ltu)) with size-constant conditioning protocol.** The thirteen boxplots in this figure are based on thirteen models of morphological differentiation, listed on the left. Each boxplot represents measurements of the resolvable proportion of clades for 100 simulations performed for each differentiation pattern under incomplete sampling, with a sampling rate of 0.1 per Ltu. Simulations were conditioned to have one hundred taxa on average at this sampling rate, under the size-constant protocol discussed in the methods. Compare to figure 4, which depicts the same analyses under the clade-constant protocol.
(TIF)

Figure S3 **Simulations of resolvability under incomplete sampling (rate of 0.01 per Ltu)) with size-constant conditioning protocol.** The thirteen boxplots in this figure are based on thirteen models of morphological differentiation, listed on the left. Each boxplot represents measurements of the resolvable proportion of clades for 100 simulations performed for each differentiation pattern under incomplete sampling, with a sampling rate of 0.01 per Ltu. Simulations were conditioned to have one hundred taxa on average at this sampling rate, under the size-constant protocol discussed in the methods. Compare to figure 5, which depicts the same analyses under the clade-constant protocol.
(TIF)
