# Supplementary figures and images for: When Can Clades Be Potentially Resolved with Morphology?
Source: PLoS One. 2013 Apr 25;8(4):e62312. doi: 10.1371/journal.pone.0062312 (PMC3636140; doi:10.1371/journal.pone.0062312)

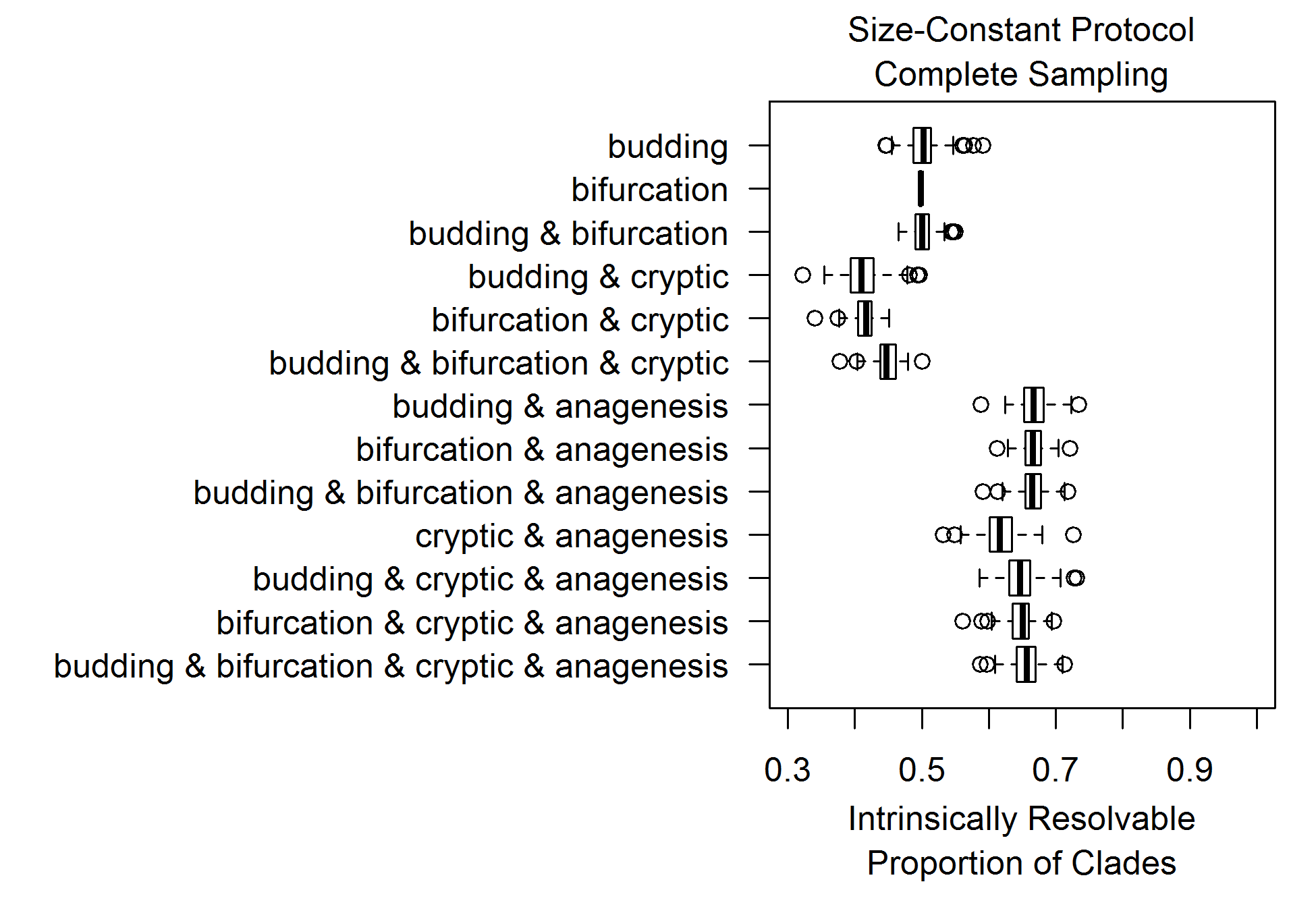

Supplement: Figure S1 — Simulations of resolvability under complete sampling with size-constant conditioning protocol. The thirteen boxplots in this figure are based on thirteen models of morphological differentiation, listed on the left. Each boxplot represents measurements of the resolvable proportion of clades for 100 simulations performed for each differentiation pattern under complete sampling. Simulations were conditioned to have one hundred taxa on average, under the size-constant protocol discussed in the methods. Compare to figure 3, which depicts the same analyses under the clade-constant protocol. (TIF) [file pone.0062312.s001.tif]

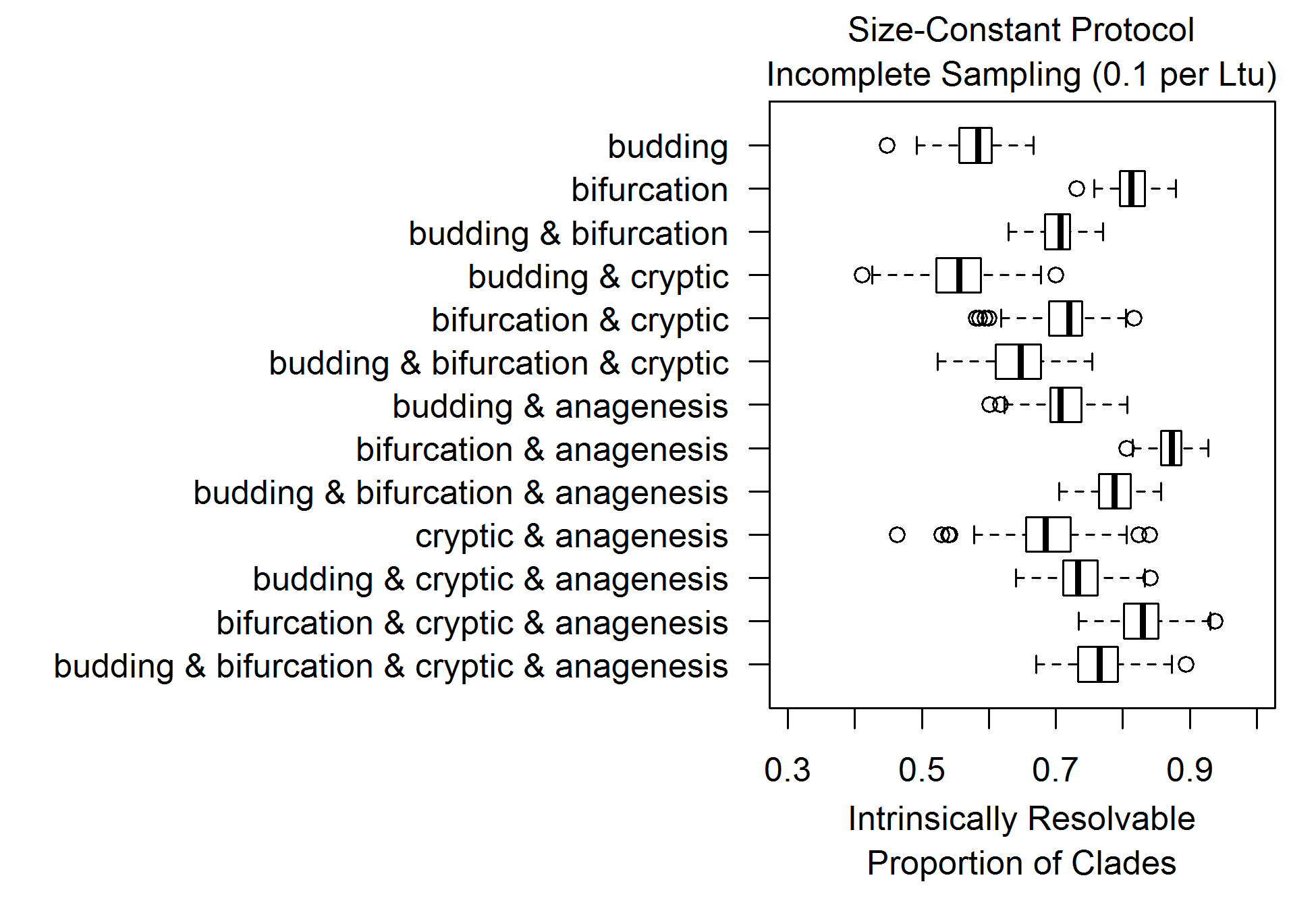

Supplement: Figure S2 — Simulations of resolvability under incomplete sampling (rate of 0.1 per Ltu)) with size-constant conditioning protocol. The thirteen boxplots in this figure are based on thirteen models of morphological differentiation, listed on the left. Each boxplot represents measurements of the resolvable proportion of clades for 100 simulations performed for each differentiation pattern under incomplete sampling, with a sampling rate of 0.1 per Ltu. Simulations were conditioned to have one hundred taxa on average at this sampling rate, under the size-constant protocol discussed in the methods. Compare to figure 4, which depicts the same analyses under the clade-constant protocol. (TIF) [file pone.0062312.s002.tif]

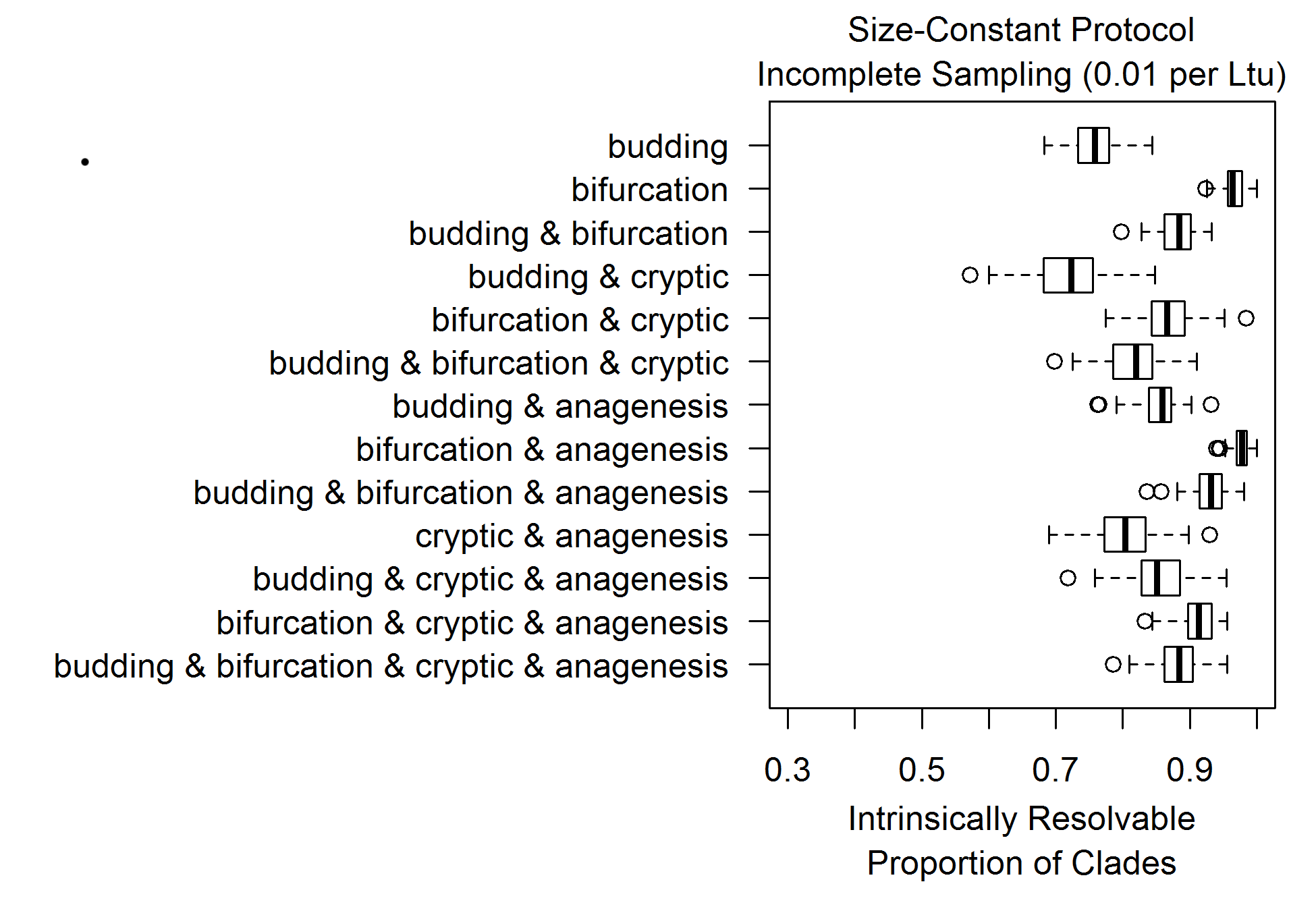

Supplement: Figure S3 — Simulations of resolvability under incomplete sampling (rate of 0.01 per Ltu)) with size-constant conditioning protocol. The thirteen boxplots in this figure are based on thirteen models of morphological differentiation, listed on the left. Each boxplot represents measurements of the resolvable proportion of clades for 100 simulations performed for each differentiation pattern under incomplete sampling, with a sampling rate of 0.01 per Ltu. Simulations were conditioned to have one hundred taxa on average at this sampling rate, under the size-constant protocol discussed in the methods. Compare to figure 5, which depicts the same analyses under the clade-constant protocol. (TIF) [file pone.0062312.s003.tif]
